# Uncommon Handlebar Hernia in an Adult Treated With Laparoscopy and Open Anatomical Repair

**DOI:** 10.7759/cureus.78437

**Published:** 2025-02-03

**Authors:** Srinivasa Swamy Bandaru, Chaitanya Garg, Maher M Milhem, Ammar Shahid Tanweer, Bashayer Alshamsi, Majd H Shaheen

**Affiliations:** 1 General Surgery and Trauma, Saqr Hospital, Emirates Health Services, Ras Al Khaimah, ARE; 2 General Surgery, RAK Medical and Health Sciences University, Ras Al Khaimah, ARE; 3 Internal Medicine, RAK Medical and Health Sciences University, Ras Al Khaimah, ARE

**Keywords:** adult individual, ct imaging, cycling accident, handlebar hernia, minimally invasive surgery, primary anatomical repair

## Abstract

Handlebar hernias, a form of traumatic abdominal wall hernia, are rare injuries resulting from blunt abdominal trauma in adults. Robust suspicion is essential for diagnosis given the subtle symptoms and intact skin.

A 48-year-old male arrived with pain and swelling on the left side of his abdomen after a low-speed bicycle crash. Imaging examinations showed a complete abdominal wall defect with herniated omental fat and unbroken skin. A diagnostic laparoscopy was carried out to exclude intra-abdominal injuries, and an open surgical repair of the hernia defect was performed. The patient recovered satisfactorily after surgery and was sent home. Handlebar hernias are frequent in children and rare in adults, usually arising from bicycle accidents. The condition frequently experiences misdiagnosis because of subtle clinical signs. This case underscores the significance of clinical suspicion and imaging, especially computed tomography (CT) scans, in the diagnosis of traumatic hernias resulting from blunt force injuries. A strong suspicion is necessary for traumatic abdominal wall hernias in cases of blunt abdominal trauma that show abdominal wall swelling and hematoma as clinical indicators. Recognizing the condition promptly and administering timely surgical intervention are crucial for effectively managing handlebar hernias to prevent complications.

## Introduction

This study was conducted in accordance with the Surgical CAse REport (SCARE) guidelines, which serve as a tool for surgeons to report their surgical cases in a standardized and systematic manner [1].

Abdominal injuries significantly impact the morbidity and mortality rates among young people globally. Traumatic abdominal wall hernia (TAWH) involves the displacement of internal organs through the abdominal wall caused by a rupture of the muscle and fascia layers, all while the skin stays unbroken [2]. A unique, specific variant of this hernia is the adult handlebar hernia (AHH), which occurs due to a low-energy direct force against bicycle* *handlebar-like items [2,3]. Identifying this condition necessitates a strong degree of suspicion since the physical examination might not distinctly show the injury, making it simple to miss the diagnosis [4,5]. Timely diagnosis of this condition is essential as it can be associated with significant visceral injuries [2]. We present a case of a 48-year-old individual who developed a traumatic left anterior abdominal wall hernia following an injury from a bicycle handlebar in a traffic accident.

## Case presentation

A 48-year-old male arrived at the emergency department within one hour of a low-speed car accident while he was cycling. The impact made the bicycle handlebar hit the left side of his abdomen. He described moderate pain centered in the left abdominal area, accompanied by swelling in that region at the time of presentation, with no other abdominal symptoms. The patient did not have any related injuries to the head, chest, pelvis, spine, or limbs. During the clinical assessment, the patient was completely awake and responsive, displaying a Glasgow Coma Scale (GCS) score of 15/15. He exhibited a high blood pressure of 189/120 mmHg, a pulse rate of 104 beats per minute, along with a normal respiratory rate and oxygen saturation. Upon examination, he showed no additional injuries aside from slight abrasions on his left foot. His determined body mass index (BMI) was 23. The patient had a history of diabetes and had not undergone any surgeries previously.

**Figure 1:**
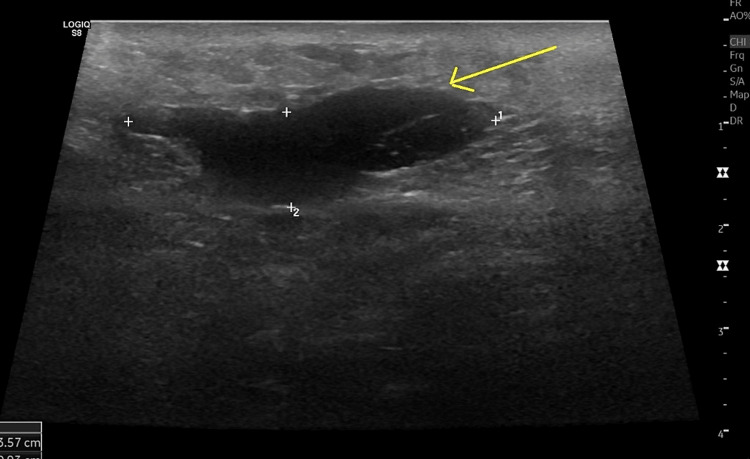
Ultrasound image showing an abdominal wall hematoma (arrow).

The abdominal examination showed a circular mass measuring 8 x 10 cm on the left upper side, clinically appearing to be a hematoma. The skin above the swelling exhibited a round reddish stain and a distinct bruise resulting from the injury inflicted by the bicycle handle (handlebar sign or "London eye sign"). There was sensitivity near the swelling, and the remainder of the abdomen was soft and relaxed with normal audible bowel sounds. The examination of the external genitalia and the inguinal area showed no abnormalities. Laboratory tests showed a normal white blood cell count, hemoglobin, and platelet count, while the C-reactive protein level was 6.4 mg/dL. Kidney and liver function tests fell within normal ranges. A focused abdominal sonography for trauma (FAST) scan and full abdominal ultrasound detected a slight accumulation of free intraperitoneal fluid and a small surface hematoma on the left lumbar region, measuring 3.5 x 1 cm (Figure 1).

**Figure 2:**
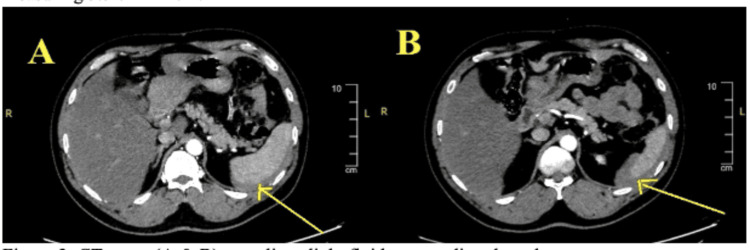
CT scans revealing intraperitoneal fluid surrounding the spleen (A and B).

**Figure 3:**
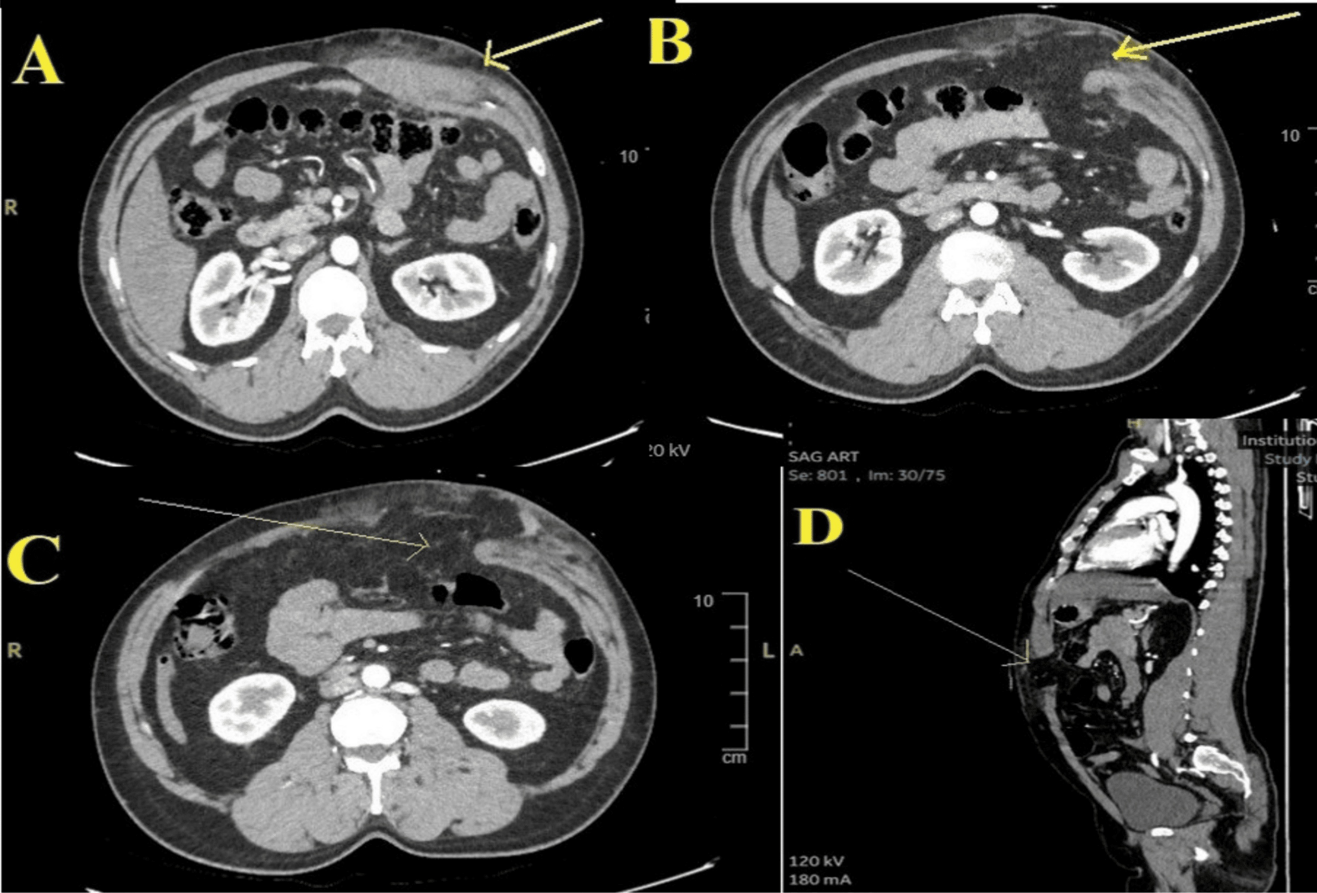
CT scans revealing abdominal wall hematoma and defect. The images show (A) hematoma in the abdominal wall muscle, axial view (arrow); (B and C) abdominal wall defect with intact skin, axial view (arrow); and (D) abdominal wall defect, sagittal view (arrow).

Additional evaluation was performed using an abdominal computed tomography (CT) scan with contrast, which showed the presence of a slight accumulation of free fluid around the spleen (Figures 2A, 2B) and right hepatic lobe, swelling in the upper section of the left rectus muscle, along with a blurry appearance of the nearby layers of subcutaneous fat and no evidence of damage to any other organs or bowels (Figure 3A). A 7 cm diameter full-thickness defect was observed in the left paramedian abdominal wall, through which a hernial sac containing fat was bulging, with intact skin (Figures 3B-3D).

Management

**Figure 4:**
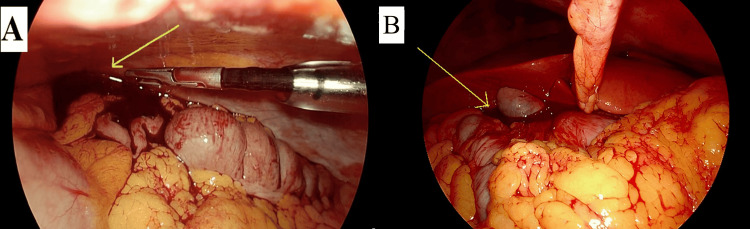
Laparoscopic surgery images revealing hemoperitoneum. The images show (A) blood in the left hypochondrium (arrow) and (B) blood around the liver (arrow).

**Figure 5:**
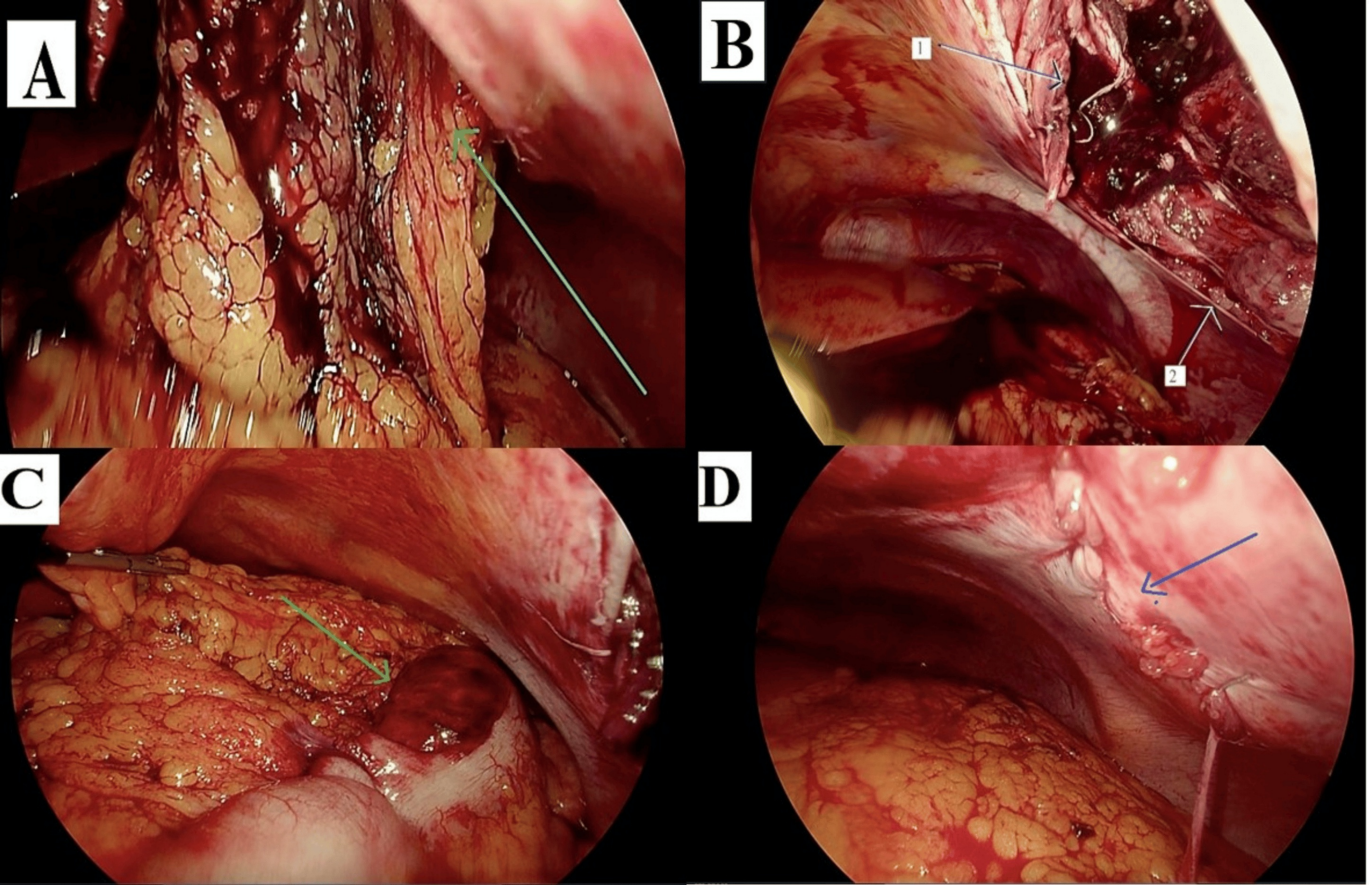
Laparoscopic surgery images revealing details of handlebar hernia findings. The images show (A) omentum protruding through the defect (arrow); (B) defect post-reduction of hernia components - (arrow 1) upper part of the hernia defect and (arrow 2) lower part of the hernia defect; (C) serosal damage of the transverse colon (arrow); and (D) anatomical repair of the hernial defect following open surgery (arrow).

The patient was admitted to the ward and started on intravenous analgesics and antibiotics. Due to the presence of free intraperitoneal blood on the CT imaging, the patient underwent diagnostic laparoscopy on the day of admission, which revealed mild hemoperitoneum mainly in the upper part of the peritoneal cavity with normal solid organs and hollow viscera (Figures 4A, 4B). It confirmed the presence of a full-thickness tear of the left-sided abdominal wall with herniation of omentum through it (Figures 5A, 5B), as well as evidence of a small area of superficial transverse colon contusion with intact viability (Figure 5C). Considering the large hernial defect and the accompanying torn muscles, it was decided to carry out an open anatomical repair. A transverse skin incision measuring 10 cm was made over the left hypochondrium at the site of the traumatic abdominal wall hernia. The abdominal wall laceration was repaired with 0 Ethibond interrupted sutures for the inner layer and 0 Prolene continuous sutures for the outer aponeurotic layer. A number 10 French units (Fr) drain was placed in the wound, the subcutaneous layer was sutured with 2-0 Vicryl, and the skin closure was done with a skin stapler. The laparoscopic repair was checked and port wounds were closed with appropriate sutures (Figure 5D).

Post-operative care and recovery

**Figure 6:**
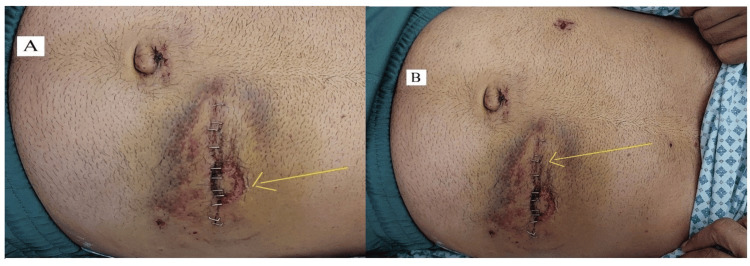
Post-operative images of the operation site. The images show (A) handlebar sign (arrow) and (B) post-operative primary repair wound demonstrating good healing (arrow).

The patient continued taking antibiotics, anti-hypertensives, and diabetes medications post-operatively. After removing drains, the patient recovered well and was discharged home on the fifth day post-operatively. He followed up at the surgery outpatient clinic two and four weeks after discharge and was doing well, with good wound healing. A well-formed healing handlebar sign was observed (Figure 6A), and there were no complications (Figure 6B).

## Discussion

Handlebar hernia is a form of traumatic abdominal wall hernia, characterized by herniation through torn muscles and fascia, with no signs of skin breach and no existing defect in the injury location [2]. They are frequent in children because of underdeveloped abdominal wall muscles and are uncommon in adults [3].

Handlebar hernias are typically classified into three types according to the proposal by Wood et al. [3]. Type I and II are categorized as small and large defects, respectively, that do not result in visceral herniation, whereas type III includes herniation of the viscera. Hernias in adults usually occur in anatomically weak areas, like the inguinal region or alongside the rectus muscle, especially in the lower abdomen, but in the present case, it was in the left hypochondrium, which is uncommon [4].

In clinical practice, the diagnosis is frequently missed and regarded as merely an abdominal wall hematoma [4]. It is sometimes associated with a distinct handlebar mark on the abdominal wall skin [4]. The Valsalva maneuver might help distinguish a hematoma from a traumatic hernia [6,7]. Imaging methods, especially ultrasound and CT scans, are essential for precise diagnosis, as shown in the present case [5,7]. Our literature review of PubMed and Google Scholar yielded around 36 reports [2-31].

Adult handlebar hernias have a strong correlation with intra-abdominal injuries, occurring in 55% of cases. These injuries typically involve mesenteric lacerations and damage to the small and large intestines (Table 1). There was one instance of injury to each of the liver [21], spleen, kidney [20], stomach [27], and appendix [15]. Our evaluation indicated that the preferred procedure was primary repair of the defect, with the option for mesh repair, primarily because of the difficulty in approximating the torn abdominal wall muscle and related necrosis (Table 1). When performed, mesh repair was often selected in the absence of related intra-abdominal injuries (except for one instance) as opposed to when such injuries were present. Nonetheless, there are no reports on the long-term follow-up regarding mesh infections and hernia recurrence [6]. Conservative management was recommended in just two instances where the injury affected the lower abdominal wall, and the size of the defect was small (Table 1) [2-31].

**Table 1:** Literature review of the management of traumatic adult handlebar hernia cases (n=36). Tables 1, 2, 3 are not directly reproduced from original articles, but they were created by the authors based on data obtained from reference articles.

Parameter	Number	Primary repair	Mesh repair	Conservative	Remarks
No associated injury	16	8	6	2	One case of mesh repair at 1-year follow-up showed no recurrence [6]
With associated injuries (n=20)	Mesenteric injuries alone=7	3	4	None	No long-term follow-up for infection or recurrence
	Bowel+solid organ injuries=13	12	1	None	No long-term follow-up for recurrence or infection

In the most extensive collection of retrospective studies on TAWH, a predominance of males was observed, with a median age varying from 36 to 39 years (Tables 2, 3) [32,33]. Surgical intervention was the primary approach to management; nonetheless, there is significant variation in the repair of the abdominal wall defect across the practice guidelines of primary repair, mesh repair, and conservative treatment. There are cases of hernia recurrence reaching as high as 26% after the initial repair of TAWH [33].

**Table 2:** Retrospective review of adult traumatic abdominal wall hernias from 2002 to 2014 (n=80). ISS: injury severity score Tables 1, 2, 3 are not directly reproduced from original articles, but they were created by the authors based on data obtained from reference articles.

Male sex	Median age	Median ISS	Motor collision	Laparotomy/laparoscopy	Bowel resection	Primary hernia repair	Overall hernia recurrence
64%	36 years	22	72.5%	44%	49% of operated	29%	26%

**Table 3:** Assessment of 38,749 trauma cases for TAWH (n=64) from 2012 to 2020. TAWH: traumatic abdominal wall hernia; ISS: injury severity score Tables 1, 2, 3 are not directly reproduced from original articles, but they were created by the authors based on data obtained from reference articles.

Percentage of TAWH	Male sex	Median age	Median ISS	Emergency surgery	Bowel resections	Non-operative management	Primary repair of hernia	Mesh repair
0.17%	66%	39 years	21	42%	25%	9.4%	6 cases	10 cases

Adult handlebar hernias are rare, with few cases documented. Our review of the literature indicates a significant threshold for the initial repair of abdominal wound defects, regardless of the presence of internal injuries (Tables 1, 2). However, mesh insertion in patients with concurrent internal injuries may not be recommended, as it could be associated with higher mesh infection rates. Recurrences following the initial repair, however, can be addressed later with mesh insertion.

## Conclusions

This report underscores the crucial role of diagnostic laparoscopy in confirming the presence of internal injuries while facilitating post-repair verification of the hernia site, reducing the chances of undetected complications. The decision to perform open anatomical repair over a purely laparoscopic approach was guided by the presence of a large hernial defect and muscle tears, highlighting the importance of tailored surgical strategies. Compared to previously reported cases, the successful use of laparoscopy for diagnostic and verification purposes in this case demonstrates its utility in managing complex traumatic hernias. This study reinforces the need for vigilance in diagnosing handlebar hernias and emphasizes the value of imaging and surgical exploration in their effective management.
